# *Varicella pneumonia* in adults: 13 years' experience with review of literature

**DOI:** 10.4103/1817-1737.36551

**Published:** 2007

**Authors:** Mohammed Alanezi

**Affiliations:** *Department of Medicine, Security Forces Hospital, Riyadh 11613, P.O Box 90068, Saudi Arabia*

**Keywords:** Outcome, pneumonia, varicella

## Abstract

Pneumonia is a serious complication of varicella infection in adults. This study investigates the clinical characteristics in 19 patients admitted to our hospital with diagnosis of *Varicella pneumonia*.

**MATERIALS AND METHODS:**

A retrospective chart review study was performed in adult patients admitted with diagnosis of *Varicella pneumonia* over 13 years (1992–2005). The study documented the clinical characteristics, laboratory investigations, hospital course, complications, treatment received and the outcomes.

**RESULTS:**

Nineteen patients were identified with a mean age of 41 (±15.4). All were males except two. Eleven patients (58%) were smokers. Eleven patients (58%) had direct contact with persons with chickenpox infection. One patient had underlying chronic pulmonary disease (sarcoidosis). Sixteen patients (84%) were admitted to the intensive care unit due to respiratory failure; eight of them required mechanical ventilation. The mean duration of ICU stay was 4.4 days. All patients were treated with acyclovir and IV antibiotics. Three patients received IV steroid. There was one death.

**CONCLUSION:**

Patients with *Varicella pneumonia* are at high risk for respiratory failure and the need for mechanical ventilation. However, early implementation of supportive therapy seems to positively influence the recovery rate and outcome.

Varicella (chickenpox) is an infectious disease caused by varicella zoster virus. The incidence of chickenpox in adults has increased in recent years, with an increment in morbidity and mortality.[1] Pneumonia is the most common and the most serious complication of chickenpox infection in healthy adults. The exact frequency of pneumonia in patients with chickenpox has been difficult to determine and most published studies represent either collections of small case series or retrospective chart review over many years.[2] However, it is estimated to occur in one out of 400 cases of chickenpox infection.[3] Risk factors for developing pneumonia include impaired immune status, chronic lung diseases, being previous or current smokers, history of contact with a patient with chickenpox, as well as severity of the skin rash. The third trimester of pregnancy was shown to be associated with an increased incidence of pneumonia.[4] It is estimated that *Varicella pneumonia* carries an overall mortality rate between 10% and 33%.[5] The mortality rates approach 50% in patients who experience respiratory failure requiring mechanical ventilation.[6] The main objective of this study was to describe the clinical manifestations, hospital course, complications and rate of morbidity and mortality and variety of treatment options in all cases of *Varicella pneumonia* in adult patients.

## Materials and Methods

A retrospective chart review was conducted to collect all relevant data from adult patients (18 years old and above) with diagnosis of *Varicella pneumonia* admitted to Security Forces Hospital, a secondary care hospital in Riyadh, Saudi Arabia, from 1992 to 2005. The following information was documented: age; sex; clinical presentation; smoking history; length of stay; admission to the intensive care unit (ICU); intubation; complications; laboratory investigations; oxygen saturation; arterial partial pressure of oxygen (PaO_2_); supportive treatment including acyclovir, antibiotics and steroids; as well as outcomes. Varicella was diagnosed based on clinical findings of fever and typical characteristic vesicular skin rash. *Varicella pneumonia* was diagnosed based on development of respiratory symptoms with radiological findings of diffuse interstitial or nodular infiltrates within 10 days following the onset of clinically evident Varicella infection.

The data were entered in MS Excel and analyzed using SPSS version 10.0 statistical software. Descriptive statistics were provided for all the study and outcome variables. The categorical variables were expressed as percentages and continuous variables were expressed as mean ± standard deviation.

## Results

From 1992 till 2005, 19 patients were identified and their data analyzed. The mean age was 41 (±15.4). Seventeen patients were male (89.5%) and 2 were female (10.5%). Eleven patients (57.9%) had direct contact with persons with chickenpox infection. Eleven patients (57.9%) were current smokers at the time of admission. One patient had history of underlying pulmonary disease (sarcoidosis). The mean hospital stay was 10.5 days (±3.5) and ranged from 4-25 days. Chest roentgenograms demonstrated bilateral interstitial/nodular infiltrates in all patients. Table 1 shows the laboratory characteristics of the analyzed cases.

**Table 1:** Laboratory characteristics of 19 adult patients with *Varicella pneumonia*

Laboratory findings	Mean ± SD	Range
pH	7.41 ± 7.98	7.29-7.49
PaO_2_ mmHg	59.58 ± 8.77	39-74
PaCO_2_ mmHg	37.21 ± 13.54	29-89
Saturation in room air (%)	87± 4.27	79-92
Bicarbonate mmol/L	24.21± 2.99	20-32
Platelets (10*9/L)	163 ± 76	70-355
Sodium (mmol/L)	135 ± 4.5	126-143
White blood count (10*9/L)	8.37 ± 2.25	4-15
Hemoglobin (g/L)	150 ± 29	120-174

Mean arterial partial pressure of oxygen (PaO_2_) was 59 mmHg (±28.1) with a range from 39-74. Sixteen patients (84.2%) were admitted to ICU with mean duration of ICU stay of 4.5 days (±3.2) and range from 1-10. Eight patients required mechanical ventilation (42.1%) with mean duration of 7.0 days (±3.2) and range from 2–10 days. All patients were treated with intravenous acyclovir 10 mg/kg every 8 h for 4 to 12 days, depending on the patient's condition and empirically with broad-spectrum antibiotics. Six patients (31.6%) were treated with steroid for 1 to 5 days. Only an 82 years old lady died due to severe respiratory failure after staying for 10 days in the intensive care unit.

## Discussion

*Varicella pneumonia* carries high risk for respiratory failure and need for mechanical ventilation, with high rate of morbidity and mortality. In a study of 12 adult patients with *Varicella pneumonia* admitted to intensive care unit in Singapore, 9 patients required mechanical ventilation, with a mortality rate of 25%.[7] In Greece, a similar study showed that 9 patients out of 22 with *Varicella pneumonia* had respiratory failure requiring mechanical ventilation with frequency 40.1%. They have concluded that adult patients with primary Varicella pneumonia who are late on asking for medical support may have more severe disease and a poor outcome.[8] In a case series from South Africa that described 15 adult patients with *Varicella pneumonia* from 1983 to 1993, 4 patients required intensive care unit and no deaths were reported.[9]

In this study, a majority of the patients were male, an observation that was also made in similar studies. This may be explained by the fact that smoking is more common in males.[10] Cigarette smoking is a major risk factor for the development of *Varicella pneumonia* in adults; this was first reported in a study of 29 patients with Varicella infection, where 7 out of 19 smokers developed pneumonia, while none of the 10 nonsmokers developed pneumonia.[11] Though immunocompromise is a well-known risk factor, none of our patients was described to be immunocompromized. The patient with underlying pulmonary sarcoidosis was not on treatment at the time of presentation.

In our study, the frequency of respiratory failure requiring mechanical ventilation was 42%, a finding that indicates that patients with *Varicella pneumonia* are at high risk for respiratory failure and subsequently require mechanical ventilation. With supportive management, the majority of them recovered. The overall mortality rate was 5.3%, which is lower than the reported 10-33% in the literature.[5] Improvement in mortality is likely to be the result of several factors, including better respiratory support in ICUs, early diagnosis and institution of acyclovir therapy.[2] However, all patients in this study were treated with acyclovir, which may explain the low mortality rate.[12,13]

There are several limitations of this study; one of these is that it is a chart review conducted in a single center. Also, it is limited by the small number of patients over a long period of time. In conclusion, patients with *Varicella pneumonia* are at high risk for respiratory failure and need for mechanical ventilation. The institution of early antiviral therapy, steroids in sick patients; and early admission to critical care unit have contributed to favorable outcome and low mortality.
